# Response Calls Evoked by Playback of Natural 50-kHz Ultrasonic Vocalizations in Rats

**DOI:** 10.3389/fnbeh.2021.812142

**Published:** 2022-01-14

**Authors:** Annuska C. Berz, Markus Wöhr, Rainer K. W. Schwarting

**Affiliations:** ^1^Behavioral Neuroscience, Experimental and Biological Psychology, Faculty of Psychology, Philipps-University Marburg, Marburg, Germany; ^2^Center for Mind, Brain and Behavior, Philipps-University Marburg, Marburg, Germany; ^3^Research Unit Brain and Cognition, Laboratory of Biological Psychology, Social and Affective Neuroscience Research Group, Faculty of Psychology and Educational Sciences, KU Leuven, Leuven, Belgium; ^4^Leuven Brain Institute, KU Leuven, Leuven, Belgium

**Keywords:** ultrasonic vocalizations, animal communication, playback, stock, strain, haloperidol, Wistar, Sprague-Dawley

## Abstract

Rats are highly social animals known to communicate with ultrasonic vocalizations (USV) of different frequencies. Calls around 50 kHz are thought to represent a positive affective state, whereas calls around 22 kHz are believed to serve as alarm or distress calls. During playback of natural 50-kHz USV, rats show a reliable and strong social approach response toward the sound source. While this response has been studied in great detail in numerous publications, little is known about the emission of USV in response to natural 50-kHz USV playback. To close this gap, we capitalized on three data sets previously obtained and analyzed USV evoked by natural 50-kHz USV playback in male juvenile rats. We compared different rat stocks, namely Wistar (WI) and Sprague-Dawley (SD) and investigated the pharmacological treatment with the dopaminergic D2 receptor antagonist haloperidol. These response calls were found to vary broadly inter-individually in numbers, mean peak frequencies, durations and frequency modulations. Despite the large variability, the results showed no major differences between experimental conditions regarding call likelihood or call parameters, representing a robust phenomenon. However, most response calls had clearly lower frequencies and were longer than typical 50-kHz calls, i.e., around 30 kHz and lasting generally around 0.3 s. These calls resemble aversive 22-kHz USV of adult rats but were of higher frequencies and shorter durations. Moreover, blockade of dopamine D2 receptors did not substantially affect the emission of response calls suggesting that they are not dependent on the D2 receptor function. Taken together, this study provides a detailed analysis of response calls toward playback of 50-kHz USV in juvenile WI and SD rats. This includes calls representing 50-kHz USV, but mostly calls with lower frequencies that are not clearly categorizable within the so far known two main groups of USV in adult rats. We discuss the possible functions of these response calls addressing their communicative functions like contact or appeasing calls, and whether they may reflect a state of frustration. In future studies, response calls might also serve as a new read-out in rat models for neuropsychiatric disorders, where acoustic communication is impaired, such as autism spectrum disorder.

## Introduction

Acoustic communication among conspecifics is an important aspect of the social life of many species and often essential for maintaining stable social structures. A characteristic feature of acoustic communication in several species is its reciprocal nature where a signal emitted by the sender frequently evokes the emission of a response signal in the receiver (Seyfarth and Cheney, 2003).

Many rodent species communicate through so-called ultrasonic vocalizations (USV), i.e., within frequencies not audible for humans (Brudzynski, 2010). In juvenile and adult rats, two main types of vocalizations are typically distinguished (Brudzynski, 2013a; Wöhr and Schwarting, 2013). Vocalizations with frequencies around 22 kHz are referred to as aversive or distress calls, presumably representing a negative affective state (Blanchard et al., 1991; Fendt et al., 2018). Vocalizations with frequencies around 50 kHz are thought to represent a positive affective state usually emitted during appetitive situations like play or mating (Knutson et al., 1998; Panksepp, 2005). These appetitive calls are typically characterized by frequencies between 35 and 80 kHz and durations in a range of 10–150 ms (Burgdorf et al., 2008; Wöhr et al., 2008; Takahashi et al., 2010). Often, such 50-kHz USV are categorized and the call categories flat, step, trill, and mixed are commonly differentiated (Kisko et al., 2018). Aversive 22-kHz USV, in contrast, have been defined between frequencies of 18 and 32 kHz (Brudzynski, 2001) and within this frequency range, short (<300 ms) and long (>300 ms) calls were identified (Brudzynski et al., 1993). Long 22-kHz calls were found to be emitted during situations of external danger, such as during the presence of a predator or during predator odor exposure, and are usually associated with freezing behavior (Blanchard et al., 1991; Fendt et al., 2018; Simmons et al., 2018). Short 22-kHz USV, however, are much more ambiguous and their function has not been identified yet (Brudzynski, 2021). It was suggested that short 22-kHz USV represent internal distress without external influence, like frustration (Taylor et al., 2019). In addition, they were repeatedly reported to occur during drug withdrawal (Ma et al., 2010; Simmons et al., 2018).

The communicative functions of 22- and 50-kHz USV can be studied by means of playback experiments (Seffer et al., 2014) and it was shown that they elicit distinct behavioral responses pattern in the receiver (Wöhr et al., 2016). Playback of natural 22-kHz USV usually induces a defensive response, including avoidance behavior and behavioral inhibition (Brudzynski and Chiu, 1995; Fendt et al., 2018). Playback of natural 50-kHz USV, in contrast, evokes social approach behavior toward the sound source (Wöhr and Schwarting, 2007). At the physiological level, playback of 22- and 50-kHz USV entail to distinct alterations. While playback of 22-kHz leads to a decrease in heart rate during behavioral inhibition, heart rate is increased during social approach behavior in response to playback of 50-kHz USV (Olszyński et al., 2020). Likewise, distinct brain activation patterns are observed. Playback of 22-kHz USV induces increased activity in the amygdala (Sadananda et al., 2008; Parsana et al., 2012), whereas playback of 50-kHz USV results in an activation of the nucleus accumbens (Sadananda et al., 2008), where it causes a phasic release of dopamine (Willuhn et al., 2014).

At the behavioral level, the social approach response toward 50-kHz USV playback can be accompanied by the emission of response calls (Wöhr and Schwarting, 2007, 2009; Willadsen et al., 2014; Willuhn et al., 2014; Engelhardt et al., 2017, 2018; Berg et al., 2018, 2021; Kisko et al., 2020; Olszyński et al., 2020, 2021). Although echoing the reciprocal nature of acoustic communication and repeatedly observed in studies applying the 50-kHz USV playback paradigm, still little is known about such response calls. In previous studies, response calls toward 50-kHz USV were observed in males and females (Berg et al., 2018, 2021), albeit the emission of calls in response to 50-kHz USV playback was found to be more prominent in males than females in one study (Kisko et al., 2020). A developmental study further suggests that age is another relevant factor, with juvenile rats emitting more response calls than adult rats (Wöhr and Schwarting, 2009). Finally, prior experiences (Olszyński et al., 2021) and inter-individual differences (Engelhardt et al., 2018) were also reported to play a role. However, the function of response calls remains elusive, which is why we wanted to shed light onto the meaning and the importance of response calls in social situations like the 50-kHz USV playback.

To close this gap, we capitalized on a previously obtained large data set and analyzed USV evoked by natural 50-kHz USV playback in male juvenile rats (Berz et al., 2021). In our previous study, we showed, amongst others, that the social approach response toward 50-kHz calls is a stable phenomenon that occurs in Wistar (WI) and Sprague-Dawley (SD) rats and that it can be modulated by administration of the dopaminergic D2 receptor antagonist haloperidol (Halo; Berz et al., 2021). Here, we present three new data sets from these previous experiments. Data set 1 was comprised of WI rats exposed to 50-kHz USV playback. We analyzed it in an initial attempt to better understand the emission of response calls and to test whether response calls occur specifically in reaction toward 50-kHz USV but not noise and whether stimulus order of 50-kHz USV and noise plays a role. Data set 2 consisted of WI and SD rats and their response calls were compared to see whether there was a difference between the stocks. In the final data set 3, rats received either Halo or saline (Sal) to investigate whether Halo treatment not only affects social approach behavior but also the emission of response calls toward 50-kHz USV playback. Our comprehensive analysis approach included a detailed investigation of the temporal emission pattern and an examination of acoustic features, focusing on numbers of calls, latencies to start calling, mean peak frequencies, call durations, and frequency modulations.

## Materials and Methods

### Animals and Housing

In total, 108 experimentally naïve juvenile male rats around 5–7 weeks of age (Charles River Laboratories, Sulzfeld, Germany) were analyzed. The sample consisted of 90 Wistar (WI) wildtype rats and 18 Sprague-Dawley (SD) wildtype rats. The animals were kept in a vivarium with a 12-hour light/dark cycle with lights on at 7 am and 32–50% humidity. They were housed in groups of five to six rats in polycarbonate cages (macrolon type IV, size 380 × 200 × 590 mm with high steel covers) where food and water were provided *ad libitum.* After arrival from the breeder, the animals had seven days to acclimate to the vivarium, followed by a standardized protocol of handling for three consecutive days, each day for 5 min. The procedures had been approved by the ethical committee of the local government (Regierungspräsidium Gießen, Germany, TVA Nr. 6 35-2018).

### Overview

Response calls were analyzed in three data sets. These sets were obtained as part of a recently published study focusing on the habituation of the social approach response to repeated playback of 50-kHz USV (Berz et al., 2021). In this previous study, rats were exposed twice to playback of 50-kHz USV and their behavioral response was quantified, i.e., locomotor activity and approach behavior. Here, we now analyzed response calls evoked by playback of 50-kHz USV that were also recorded in this study. We focused on the emission of response calls during the first playback exposure because preliminary data indicate that call emission decreases with repeated playback presentations similar to social approach behavior (Berz et al., 2021). In the first data set, we analyzed response calls in WI rats (*N* = 24) and tested whether their emission occurs specifically during playback of 50-kHz USV but not noise and whether their emission depends on stimulus order. Rats were weighing 144.25 ± 1.88 g (range 128.5–164.5 g). In the second data set, we compared the production of response calls between WI rats (*N* = 18) to that of SD rats (*N* = 18). Rats were weighing 163.47 ± 2.85 g (range 138.5–205 g). In the third data set, we studied the role of the dopaminergic system in regulating the emission of response calls and compared response calls emitted by WI rats systemically treated with the dopaminergic D2 receptor antagonist Halo (*N* = 24) and saline treated controls (*N* = 24). Rats were weighing 189.57 ± 2.95 g (range 147.5–233 g).

### Drug Treatment

In the third data set, rats received the dopaminergic D2 receptor antagonist Halo (0.5 mg/kg; Haldol, Janssen, Belgium) or saline (Sal, 0.9% NaCl solution, Braun, Germany). The ip injection took place 60 min before the start of the playback experiment and during the time between the injection and the playback experiment, rats were kept singly (in a small cage with bedding and water *ad libitum*) in a dark room (according to Tonelli et al., 2017).

### 50-kHz Ultrasonic Vocalizations Playback: Setup

As experimental setups, an eight-arm radial maze (data sets 1 and 2) and a squared platform (data set 3), each elevated 52 cm above the ground, were employed. On two opposite sides of the given apparatus, an ultrasonic speaker (ScanSpeak, Avisoft Bioacoustics, Berlin, Germany) and an ultrasonic condenser microphone (CM16, Avisoft Bioacoustics) were placed 20 cm away from the end of the arm or platform. Only one of the speakers was active, whereas the other one served as a visual control. Experiments were conducted under red light (∼10 lux).

### 50-kHz Ultrasonic Vocalizations Playback: Acoustic Stimuli

We presented two types of acoustic stimuli: (A) 50-kHz USV recorded from an adult male WI rat (ca. 350 g) during exploration of a cage containing scents from a recently removed cage mate (for details see Wöhr et al., 2008). This recording was composed of a sequence of 3.5 s with 13 different 50-kHz calls (total calling time 0.9 s) presented in a loop (for details see Wöhr and Schwarting, 2007). The peak amplitude was 70 dB (measured from a distance of 40 cm), being in the typical range of 50-kHz USV (Kisko et al., 2020). (B) Time- and amplitude-matched noise was generated with SASLab Pro (Version 4.2, Avisoft Bioacoustics) by replacing each 50-kHz call by noise with matching duration and amplitude modulation. Accordingly, each noise playback series had the same temporal pattern and all call features were identical, except that the sound energy was not in a certain frequency range as in the natural 50-kHz USV playback (for details see Wöhr and Schwarting, 2012). The acoustic stimuli were presented *via* an ultrasonic speaker (ScanSpeak, Avisoft Biosacoustics) with a frequency range of 1–120 kHz and a flat frequency response (±12 dB) between 15 and 80 kHz. Sounds were played *via* a portable ultrasonic power amplifier with a frequency range of 1–125 kHz (Avisoft Bioacoustics) and *via* an external sound card with a sampling rate of 192 kHz (Fire Wire Audio Capture FA-101, Edirol, London, United Kingdom).

### 50-kHz Ultrasonic Vocalizations Playback: Paradigm

At the beginning of the playback experiment, rats were placed individually in the center of the eight-arm radial maze (data sets 1 and 2) or the squared platform (data set 3). After an initial habituation period of 15 min, the first playback presentation of 5 min duration commenced. The second playback presentation of 5 min duration followed after an inter-stimulus interval of 10 min. Acoustic stimuli (i.e., 50-kHz USV, noise) were presented in a counterbalanced manner. The trial ended with a post-stimulus interval of 10 min. The whole paradigm lasted 45 min.

### Recording and Analysis of Response Calls

For recording response calls emitted by the given experimental rat, two ultrasonic microphones were placed symmetrically on two sides of the maze (data sets 1 and 2) or the platform (data set 3) next to the speakers. They were connected *via* an UltraSoundGate 416H USB audio device (Avisoft Bioacoustics) to a computer, where acoustic data were recorded with a sampling rate of 250 kHz (16-bit format; recording range 0–125 kHz) using RECORDER USGH (Avisoft Bioacoustics). For acoustical analysis, recordings were transferred to DeepSqueak (version 2.6.1, Windows standalone), a deep learning-based system for detection and analysis of USV (Coffey et al., 2019). Recorded files were converted into high-resolution spectrograms and were analyzed using the pre-trained automated ‘‘short rat call network V2.’’ The settings for call detection were ‘‘high recall,’’ with an overlap of 0.001 s. This setting was chosen because it minimizes the possibility that a call is missed, albeit at the cost of false positives by including noise. Therefore, a custom trained network for denoising was applied afterward. The detected events were then transferred into the DeepSqueak Screener (Fork on GitHub by L. Lara-Valderrábano and R. Ciszek: 10.5281/zenodo.3690137),^1^ where the files were reviewed and denoised again manually by an experienced observer accepting (response calls) or rejecting (noise or playback calls) events. All response calls, irrespective of frequencies and durations, were counted. For later analysis, response calls during the 5 min before, during, and after the playback presentations (50-kHz USV or noise) were taken into account (referred to as stimulus phase). Outside this time window, calls occurred rarely. Acoustic features, i.e., call duration, peak frequency, and frequency modulation (difference between highest and lowest frequency), were defined and analyzed as described previously (Kisko et al., 2018). For classifying response calls, we applied previously established frequency thresholds (Brudzynski, 2001). Calls with frequencies higher than 32 kHz were classified as 50-kHz USV and calls below 32 kHz were defined as 22-kHz USV.

### Recording and Analysis of Overt Behavior

As pointed out above, the behavioral data (locomotion, approach) were part of a recently published study focusing on the habituation of the social approach response to playback of 50-kHz USV (Berz et al., 2021). Here, we reconsidered these data in the context of the new data on response calls in order to address the question whether locomotor activity and approach behavior evoked by playback of 50-kHz USV are associated with the emission of response calls. Briefly, overt behavior was recorded and analyzed using EthoVision XT (Version 13, Noldus, The Netherlands). Locomotion was measured by the distance traveled. For quantifying approach behavior on the maze (data sets 1 and 2), the numbers of entries into the three arms proximal and distal to the active speaker and the time spent thereon were measured. For quantifying approach behavior on the platform (data set 3), it was virtually divided into 25 equal quadrants, with the six quadrants close to the active speaker serving as proximal zone, while the six quadrants close to the inactive speaker were defined as distal zones. Entries and time spent in these zones were measured (for details see Berz et al., 2021).

### Statistical Analysis

Analyses of variance (ANOVAs) for repeated measurements were calculated with the between-subject factors playback order (50-kHz USV first vs. second), stocks (WI vs. SD), or drug treatment (Halo vs. Sal), and the within-subject factors stimulus phase (5 min before, during, or after playback) and playback stimulus (50-kHz USV or time- and amplitude-matched noise). This was followed by two-tailed *t*-tests for comparing individual experimental groups. The ratio between calling and non-calling rats was evaluated by a χ^2^-test (calculated using https://www.socscistatistics.com/tests/chisquare2/default2.aspx). Approach behavior was quantified by subtracting the times spent on proximal arms (or in proximal zones) before the 5 min of 50-kHz USV playback from the time spent there during the 5 min of playback. The same was done with the entries into proximal arms or zones. Pearson correlation coefficients (bivariate) were calculated for the correlation between numbers of emitted calls and approach behavior. For testing a possible correlation with locomotor behavior, locomotion (distance traveled in cm) during the 5 min before playback were subtracted from that during the 5 min during playback. This number was then correlated with the numbers of response calls emitted using the Pearson correlation coefficient. For general locomotor activity correlations, the distance traveled during the initial 15-min habituation period were taken into account. All *t*-tests, ANOVAs, and correlations were calculated with IBM SPSS Statistics (version 25). Graphs were made using GraphPad Prism (version 8). Data are represented as means ± SEM (standard error of mean). A *p*-value of < 0.050 was considered statistically significant.

## Results

### Data Set 1: Response Calls

#### Call Numbers and Latencies

**FIGURE 1:**
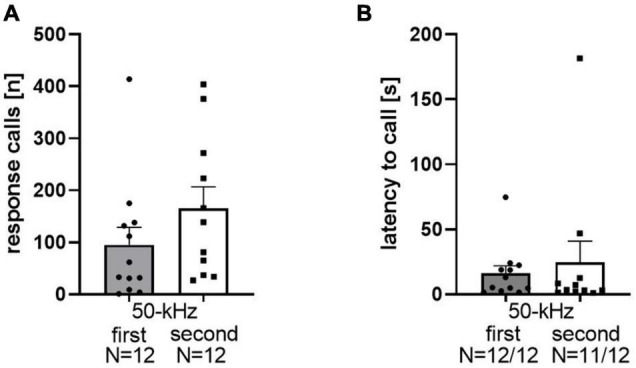
Total numbers of response calls emitted **(A)** for playback of 50-kHz as the first stimulus [95.25 ± 33.62 (mean ± SEM)] or as the second stimulus (152 ± 40.07) of WI rats. Latencies after stimulus onset **(B)**: 50-kHz first: 16.04 ± 5.67; 50-kHz second: 24.67 ± 16.19.

Playback of 50-kHz USV induced response calls in the majority of WI rats. Among the 24 rats of data set 1, 23 of them emitted response calls. The mean number of response calls was 123.5 ± 26.21, ranging between 0 and 414 calls in total per rat (Figure 1A). During the 5 min before 50-kHz USV playback, no calls were emitted. The occurrence of response calls was not dependent on whether 50-kHz USV were presented as the first or the second stimulus (*t*_22_ = 0.82, *p* = 0.21). Importantly, high levels of response calls were emitted specifically in reaction toward playback of 50-kHz USV but not noise, irrespective of whether 50-kHz USV were presented as the first (*t*_11_ = 2.8, *p* = 0.017) or the second stimulus (*t*_11_ = 4.013, *p* = 0.002; Figure 1A). The latency to start calling after onset of 50-kHz USV was 20.17 ± 88.17 s (Figure 1B). Stimulus order did not affect call latency (*t*_21_ = 0.52, *p* = 0.61). Therefore, we abstained from differentially considering stimulus order further in all following analyses.

### Data Set 2: Stock Differences

#### Call Numbers and Latencies

**FIGURE 2:**
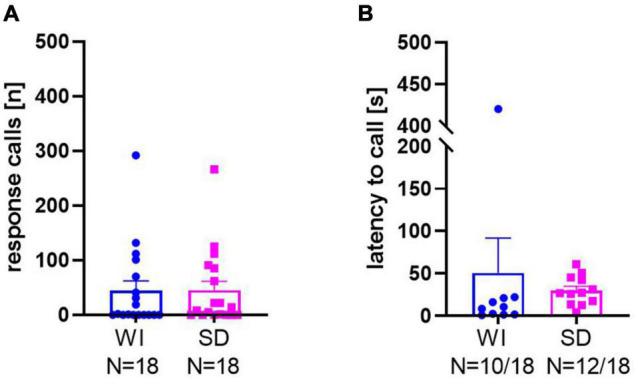
Total numbers of response calls **(A)** and latencies to call **(B)** in Wistar (WI) and Sprague-Dawley (SD) rats.

Consistent with data set 1, response calls were seen in the majority of rats in data set 2 focusing on possible stock differences between WI and SD rats. From the two different stocks, 10 out of the 18 WI rats emitted calls in response to 50-kHz USV playback and 12 out of 18 SD rats did. The ratios between calling and non-calling rats did not differ between stocks (*x*^2^_1, 36_ = 0.468, *p* = 0.49). Likewise, the mean numbers of response calls (Figure 2A; *t*_34_ = 0.032, *p* = 0.975; WI: 44.39 ± 17.81; SD: 45.17 ± 16.45) as well as the latencies to start calling (Figure 2B; *t*_20_ = 0.547, *p* = 0.590; WI: 50.56 ± 41.16 s; SD: 29.88 ± 4.93 s) did not differ between WI and SD. In both stocks, high levels of response calls were exclusively evoked by playback of 50-kHz USV, while response calls rarely occurred during noise playback (WI: *t*_17_ = 2.717, *p* = 0.015; SD: *t*_17_ = 2.727, *p* = 0.014).

### Data Set 1 and 2: Detailed Analyses

#### Temporal Emission Pattern

**FIGURE 3:**
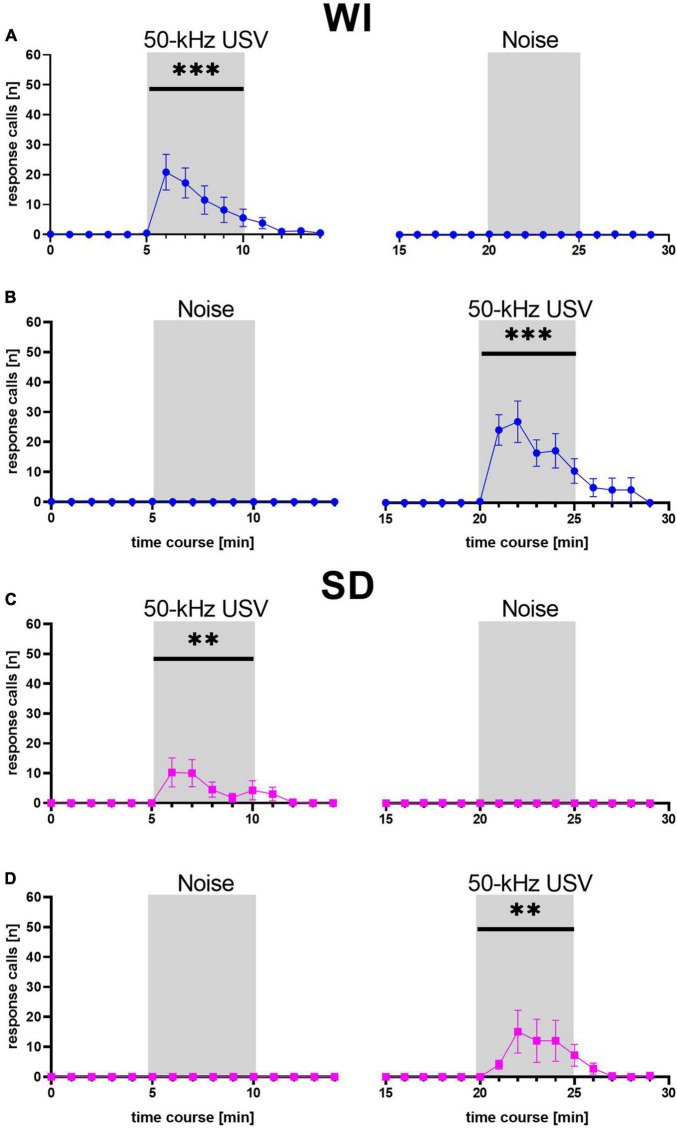
Mean numbers (±SEM) of response calls emitted during each minute of WI (blue dots; **A,B**) and SD (magenta squares; **C,D**) rats. **p < 0.01, ***p < 0.001.

We next pooled the data sets 1 and 2 and performed more detailed analyses. First, a detailed temporal analysis revealed that the emission of response calls was strongly dependent on stimulus (*F*_1, 58_ = 21.260, *p* < 0.001) and stimulus phase (*F*_2, 116_ = 21.120, *p* < 0.001), with an interaction between stimulus and stimulus phase (*F*_2, 116_ = 21.002, *p* < 0.001), while stock had no major impact (stock: *F*_1, 58_ = 2.311, *p* = 0.134; stock × stimulus: *F*_1, 58_ = 2.253, *p* = 0.139; stock × stimulus phase: *F*_2, 116_ = 2.308, *p* = 0.104; stock × stimulus × stimulus phase: *F*_1, 116_ = 2.290, *p* = 0.106; Figure 3). Specifically, playback of 50-kHz USV but not noise led to a prominent increase in response calls, which occurred during the 5 min of 50-kHz USV playback and up to 5 min thereafter. The peak of vocalization typically occurred in the second or third minute after 50-kHz USV playback onset. With onset of the 50-kHz USV playback, the numbers of emitted response calls increased significantly in WI (*F*_1, 41_ = 27.940, *p* < 0.001) and SD rats (*F*_1, 17_ = 7.436, *p* = 0.014). After that, calling rate decreased to zero at the latest 5 min after the playback had ended. In both stocks, substantial calling only occurred in response to 50-kHz USV playback and not in response to noise, reflecting high specificity of response call emission (WI: *F*_1, 41_ = 25.387, *p* < 0.001; SD: *F*_1, 17_ = 7.538, *p* = 0.014). Furthermore, the call emission sequence showed that most animals started calling with higher frequencies around 50 kHz and quickly changed to emit calls of frequencies around 22 kHz (Supplementary Figure 1A).

#### Response Call Features

Secondly, detailed analyses of acoustic features revealed that the calls in response to 50-kHz USV playback were heterogeneous since they were characterized by a large variability in acoustic features and shapes. Both, WI and SD rats emitted calls below and above 32 kHz. These calls had rather different durations and shapes, and the temporal spaces between them varied substantially.

**FIGURE 4:**
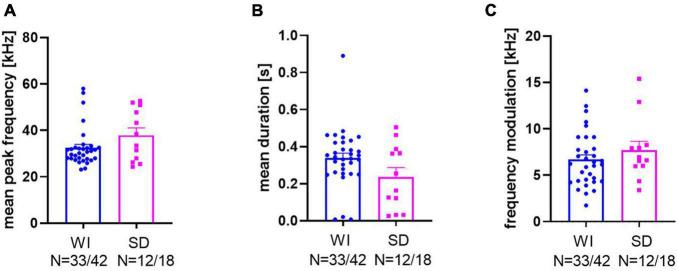
Bar graphs and individual data points of mean peak frequency **(A)**, mean duration **(B)**, and frequency modulation **(C)** of WI (blue dots) and SD rats (magenta squares).

**FIGURE 5:**
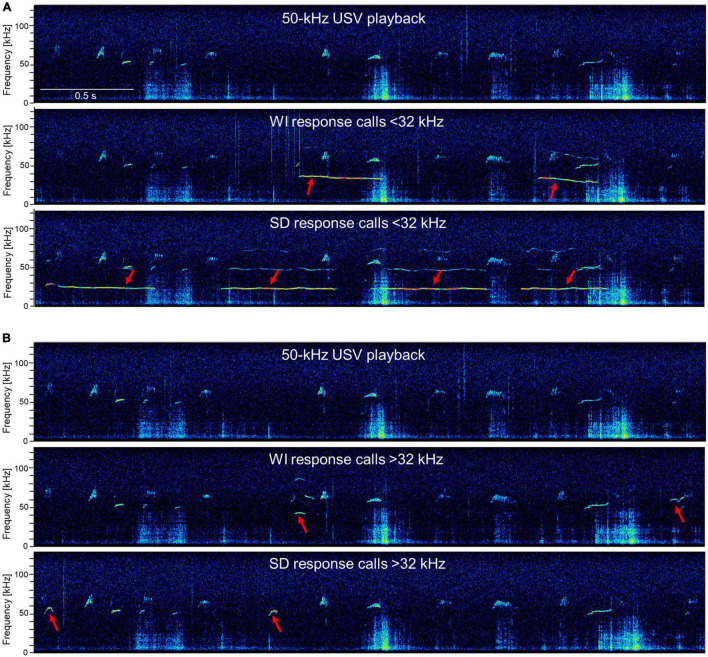
Exemplary response calls during 50-kHz USV playback. The first picture is always the 50-kHz USV playback sequence and the following pictures show response calls in addition to the 50-kHz USV playback sequence (red arrows) < 32 kHz **(A)** or > 32 kHz **(B)** of WI and SD rats. Exemplary high-resolution spectrograms (frequency resolution 488 Hz; time resolution 0.512 ms) were generated with SASLab Pro software 5.2.09 (Avisoft Bioacoustics) *via* fast Fourier transformation (512 FFT length, 100% frame, Hamming window, and 75% time-window overlap).

For a further quantification of the response calls, mean peak frequencies, mean call durations, and mean frequency modulations were quantified (Figure 4; for examples of response calls, see Figure 5). Peak frequencies of WI rats (32.48 ± 1.46 kHz) and SD rats (37.82 ± 3.2 kHz; Figure 4A) did not differ significantly from each other (*t*_15_._82_ = 1.52, *p* = 0.149). Call durations of WI rats (0.34 ± 0.03 s) tended to be longer than those of SD rats (0.24 ± 0.05 s; t_43_ = 1.859, *p* = 0.07). Frequency modulations did not differ between stocks (*t*_43_ = 0.98, *p* = 0.33; WI: 6.68 ± 0.51 kHz; SD: 7.68 ± 0.97 kHz).

**FIGURE 6:**
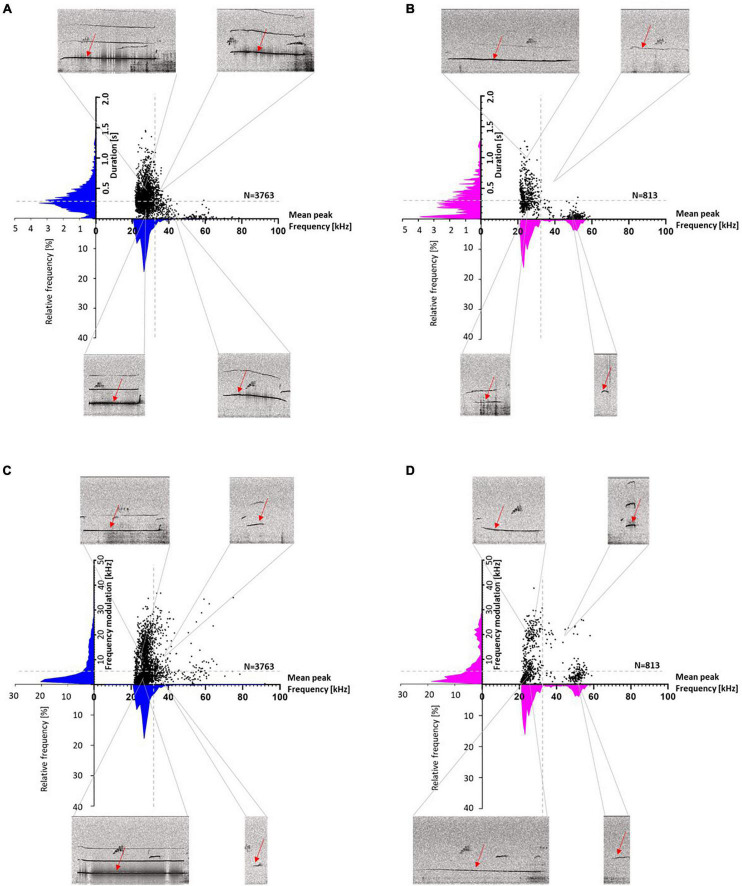
Wistar (WI) **(A,C)** and Sprague-Dawley (SD) rats **(B,D)** scatter plots with histograms (blue for WI and magenta for SD rats) of duration or frequency modulation vs. mean peak frequency. Duration is divided into <> 0.3 s (**A,B**: horizontal gray dashed lines), frequency modulation is divided into <> 5 kHz (**C,D**: horizontal gray dashed lines) and mean peak frequencies are divided into <> 32 kHz (vertical gray dashed line). For each section, an exemplary call with the respective parameters is shown (red arrows).

To visualize the different call parameters and the distribution of individual calls, scatter plots for either call durations or frequency modulations were plotted vs. peak frequencies (Figure 6). This analysis showed that most calls were below 32 kHz, with durations above and below 0.3 s. Frequency modulations were mainly below 5 kHz. The main distribution of the calls was around mean peak frequencies below 32 kHz in both stocks, but in SD rats also another distribution peak occurred around 50 kHz, with call durations typically shorter than 0.3 s and frequency modulations below 5 kHz (Figures 6B,D).

Next, we quantified call numbers depending on acoustic call features and divided response calls into those with mean peak frequencies below or above 32 kHz, durations shorter or longer than 0.3 s, and frequency modulations below or above 5 kHz (Table 1). This analysis showed that in both stocks the majority of response calls was below 32 kHz. Considering durations, most calls were shorter than 0.3 s, particularly in SD rats. Frequency modulations were mainly below 5 kHz. When comparing the percentages of calls with mean peak frequencies below 32 kHz among stocks, WI rats were found to have higher percentages of calls below 32 kHz (*t*_43_ = 2.137, *p* = 0.038). Considering percentages of calls with durations below 0.3 s, stocks did not differ (*t*_43_ = −1.95, *p* = 0.058). The same was true for frequency modulations. Similar percentages of calls were emitted with modulations below 5 kHz in both stocks (*t*_43_ = 0.173, *p* = 0.864).

**TABLE 1:** Scatter plot distributions for Wistar (WI) and Sprague-Dawley (SD) rats.

WI *N* = 33/42			Mean peak frequency		
			=32 kHz	> 32 kHz	Total calls
	Total numbers (percentages) means ± SEM		3,328 (88.44%) 69.34 ± 5.61	435 (11.56%) 30.66 ± 5.61	3,763 (100%)
Duration	<0.3 s	1,936 (51.44%) 48.69 ± 4.5	1,644 (43.7%)	292 (7.8%)	
	>0.3 s	1,827 (48.56%) 51.34 ± 4.5	1,684 (44.8%)	143 (3.8%)	
Modulation	<5 kHz	2,272 (60.38%) 51.62 ± 4.18	2,104 (55.9%)	168 (4.5%)	
	>5 kHz	1,491 (39.62%) 48.38 ± 4.18	1,224 (32.5%)	267 (7.1%)	

**SD** ***N* = 12/18**			**Mean peak frequency**		
			=**32 kHz**	**>32 kHz**	**Total calls**

			599 (73.7%) 44.29 ± 11.95	214 (26.3%) 55.71 ± 11.95	813 (100%)
Duration	<0.3 s	479 (58.9%) 66.79 ± 9.2	273 (33.6%)	206 (25.3%)	
	>0.3 s	334 (41.1%) 33.21 ± 9.2	326 (40.1%)	8 (1%)	
Modulation	<5 kHz	464 (57.1%) 50.21 ± 7.62	324 (39.9%)	140 (17.2%)	
	>5 kHz	349 (42.9%) 49.79 ± 7.62	275 (33.8%)	74 (9.1%)	

*Mean peak frequencies < or > 32 kHz, Durations = or > 0.3 s, frequency modulations = or > 5 kHz.*

**FIGURE 7:**
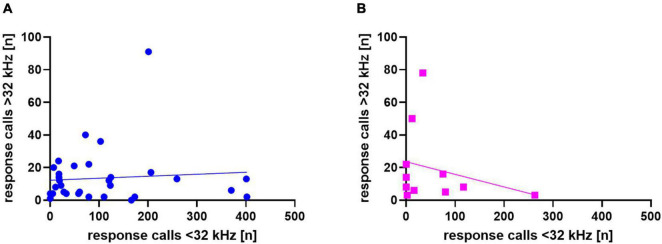
Correlation between calls <32 kHz and >32 kHz for Wistar (WI) **(A)** and Sprague-Dawley (SD) **(B)** rats. Each data point represents response calls below and above 32 kHz of one animal.

In addition, we asked whether response calls below or above 32 kHz were related to each other in individual animals (Figure 7), but did not find significant correlations between the two in WI (*r* = 0.08, *p* = 0.66) or SD rats (*r* = −0.26, *p* = 0.44).

#### Relationships Between Response Calls and Playback-Induced Approach

Thirdly, we asked whether the emission of the response calls was correlated with social approach behavior evoked by playback of 50-kHz USV. As stated in the Introduction, the present response call data sets were obtained in a study where social approach behavior evoked by 50-kHz USV playback was examined (Berz et al., 2021). In that study, approach behavior was quantified by subtracting the time spent on the proximal arms (i.e., close to the speaker) before playback from the time spent thereon during the presentation of 50-kHz USV. The same was done for the proximal entries (see detailed analysis in Berz et al., 2021). These numbers were now correlated with the total amount of response calls evoked by playback of 50-kHz USV to see whether social approach behavior was related to the emission of response calls across individual rats. In WI rats, this tended to be the case. The more time the rats spent close to the active speaker, the more calls in response to 50-kHz USV playback they tended to emit (*r* = 0.314, *p* = 0.075). A more prominent correlation was evident in SD rats, where social approach and the emission of response calls were strongly associated (SD: *r* = 0.662, *p* = 0.019). No such correlations were found with respect to proximal arm entries (WI: *r* = 0.01, *p* = 0.952; SD: *r* = −0.017, *p* = 0.948). To test whether these correlations were only a byproduct of locomotor activity during playback, the total numbers of response calls were correlated with the degree of locomotor activation using the distance traveled during playback in comparison to the distance traveled before playback. Neither in WI nor SD rats a correlation was found (*r* = 0.065, *p* = 0.681; *r* = 0.151, *p* = 0.551, respectively). Also, the numbers of response calls were not correlated with locomotor activity during the first 15 min on the maze as a measure of general locomotor activity (WI: *r* = 0.031, *p* = 0.864; SD: *r* = 0.187, *p* = 0.540).

### Data Set 3: Effects of Drug Treatment

#### Call Numbers and Latencies

**FIGURE 8:**
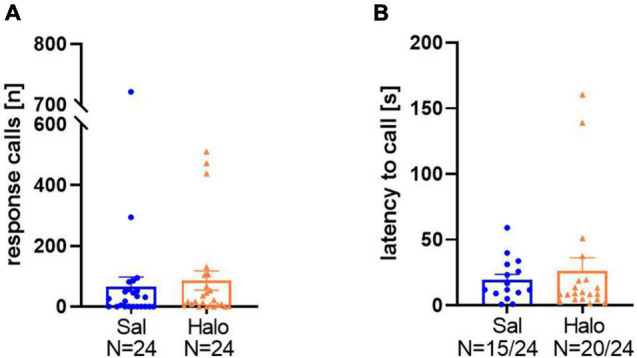
Total numbers of response calls **(A)** and latencies to call **(B)** in Sal- and Halo-treated rats. Data are presented as individual results and as means ± SEM.

In the third data set, rats were treated either with the dopaminergic D2 receptor antagonist Halo or saline as a control. The pharmacological treatment had no prominent effect on the emission of response calls and the proportion of vocalizing rats (saline: 15 out of 24, Halo: 20 out of 24) did not differ between Sal and Halo (*x*^2^_1, 48_ = 2.64, *p* = 0.104). Moreover, treatment did not affect response call numbers (*t*_46_ = 0.465, *p* = 0.644; Figure 8A; Sal: 66.5 ± 31.18; Halo: 86 ± 31.53) and latencies to start calling (*t*_33_ = 0.578, *p* = 0.567; Figure 8B; Sal: 19.41 ± 4.18 s; Halo: 26.33 ± 9.86 s).

#### Temporal Emission Pattern

**FIGURE 9:**
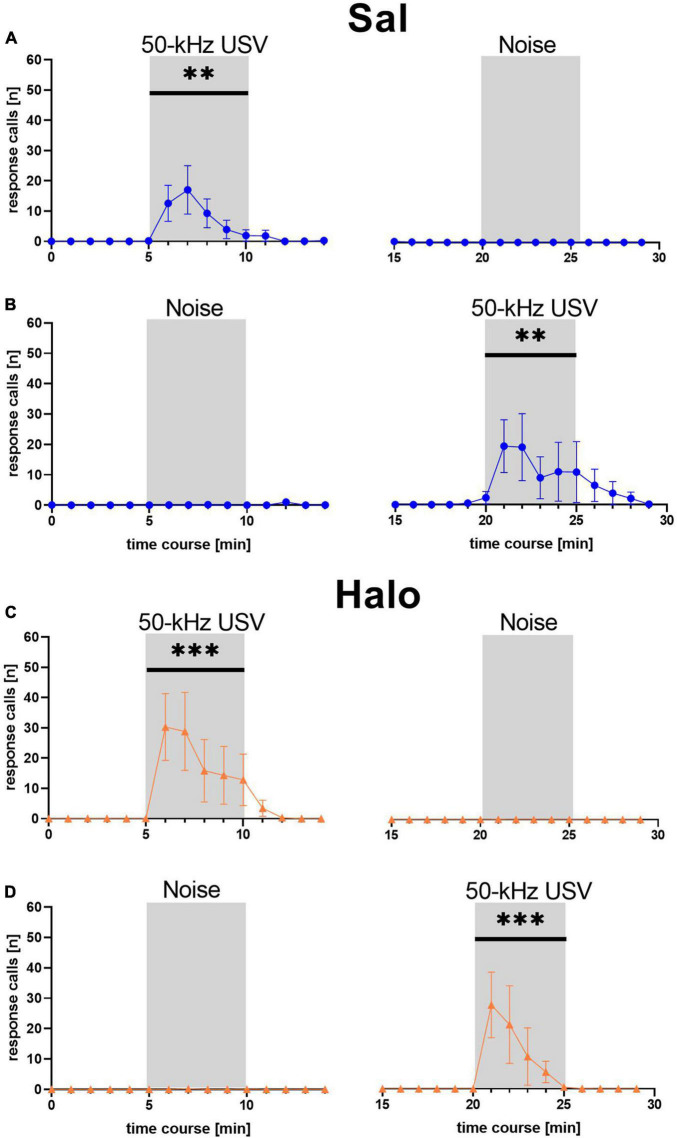
Mean number of response calls emitted during each minute of Sal- (blue; **A,B**) and Halo-treated (orange; **C,D**) rats. Most calls were emitted during 50-kHz USV stimulus and almost no calls were emitted during noise. **p < 0.01, ***p < 0.001.

Similar to the previous data sets 1 and 2, the emission of response calls was strongly dependent on stimulus (*F*_1, 46_ = 11.771, *p* = 0.001) and stimulus phase (*F*_2, 92_ = 14.443, *p* < 0.001), with an interaction between stimulus and stimulus phase (*F*_2, 92_ = 14.373, *p* < 0.001), while treatment had no major impact (treatment: *F*_1, 46_ = 0.194, *p* = 0.662; treatment × stimulus: *F*_1, 46_ = 0.232, *p* = 0.632; treatment × stimulus phase: *F*_2, 92_ = 0.842, *p* = 0.434; treatment × stimulus × stimulus phase: *F*_1, 92_ = 0.797, *p* = 0.454; Figure 9). Specifically, as in the previous data sets 1 and 2, playback of 50-kHz USV but not noise led to a prominent increase in response calls, which occurred during the 5 min of 50-kHz USV playback and up to 5 min thereafter. The peak was again typically seen during the second or third minute after 50-kHz USV playback onset. With onset of 50-kHz USV playback, the numbers of emitted response calls increased significantly in rats treated with Sal (*F*_1, 23_ = 6.443, *p* = 0.018) but also in rats treated with Halo (*F*_1, 23_ = 8.068, *p* = 0.009). After that, calling rate decreased to zero at the latest 5 min after playback had ended. Substantial calling only occurred in response to 50-kHz USV and not in response to noise and was therefore specific to the 50-kHz USV playback in both treatment groups (Sal: *F*_1, 23_ = 4.687, *p* = 0.041; Halo: *F*_1, 18_ = 7.613, *p* = 0.013). Furthermore, the call emission sequence showed that most animals started calling with higher frequencies around 50 kHz and quickly changed to emit calls of frequencies around 22 kHz (Supplementary Figure 1B).

#### Response Call Features

**FIGURE 10:**
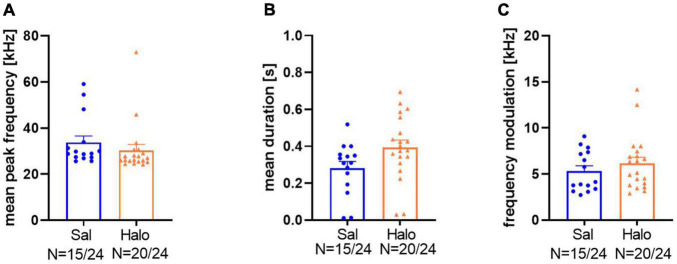
Call parameters of Sal- (blue) and Halo-treated (orange) rats for mean peak frequency **(A)**, mean duration **(B)**, and frequency modulation **(C)**.

For a further characterization of response calls in the third data set, their mean peak frequencies, durations, and frequency modulations were analyzed. Sal-treated animals had peak frequencies around 33.76 ± 2.8 kHz, which was not significantly different from Halo-treated animals (30.89 ± 2.49 kHz; *t*_33_ = 0.898, *p* = 0.376; Figure 10A). Call durations in controls were 0.282 ± 0.036 s, which was significantly shorter than those of Halo-treated rats (0.395 ± 0.039 s; *t*_33_ = 2.048, *p* = 0.049, Figure 10B). Frequency modulation did not differ between treatment groups and Sal-treated rats called with a frequency modulation of 5.33 ± 0.56 kHz compared to 6.16 ± 0.66 kHz in HALO-treated rats (*t*_33_ = 0.919, *p* = 0.365; Figure 10C).

**FIGURE 11:**
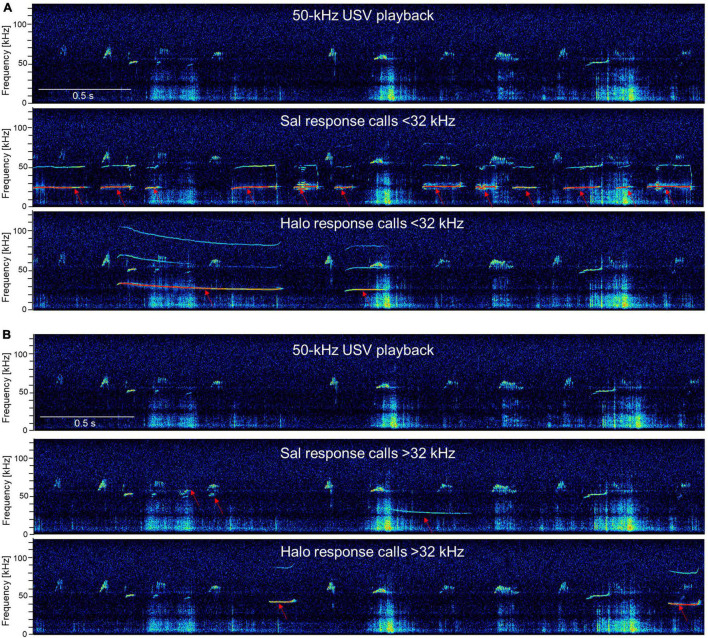
Exemplary response calls during 50-kHz USV playback. The first picture is always the 50-kHz USV playback sequence and the following pictures show response calls in addition to the 50-kHz USV playback sequence (red arrows) <32 kHz **(A)** or >32 kHz **(B)** of Sal- and Halo-treated rats. Note that the calls depicted for Sal- or Halo-treated rats are not specific to the treatment groups and calls were descriptively similar in all groups.

**FIGURE 12:**
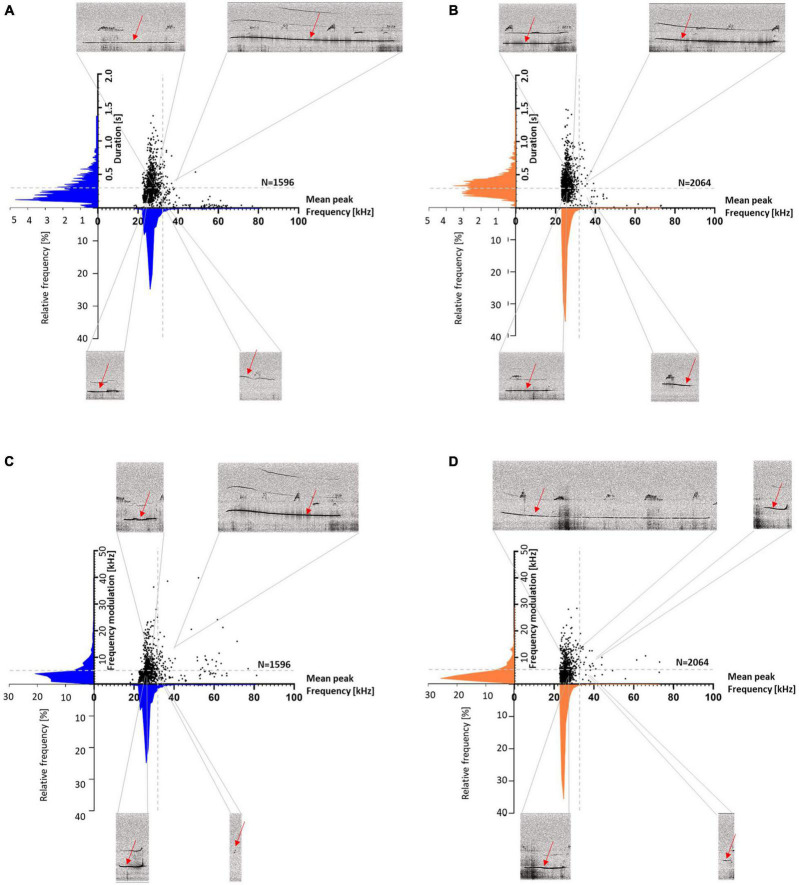
Sal- **(A,C)** and Halo-treated rats **(B,D)** scatter plots with histograms (blue for Sal- and orange for Halo-treated rats) of duration or frequency modulation vs. mean peak frequency. Duration is divided into <> 0.3 s (**A,B**: horizontal gray dashed lines), frequency modulation is divided into <> 5 kHz (**C,D**: horizontal gray dashed lines) and mean peak frequencies are divided into <> 32 kHz (vertical gray dashed line). For each section, an exemplary call with the regarding parameters is shown (red arrows).

The response calls were various in shape and differed in call parameters (for examples of response calls, see Figure 11). For better visualization of the different call parameters and the distribution of the individual calls, scatter plots for either call durations or frequency modulations were plotted vs. peak frequencies (Figure 12). The accompanying histograms show the main distribution at mean peak frequencies around 25 kHz in both treatment groups; meaning that the majority of calls were below 32 kHz. Especially in Halo-treated rats, few calls were above 32 kHz. Call durations were as well above as below 0.3 s in Sal- and Halo-treated rats. Frequency modulation was mainly below 5 kHz.

Next, we again quantified call numbers depending on acoustic call features and divided response calls into those with mean peak frequencies below or above 32 kHz, durations shorter or longer than 0.3 s, and frequency modulations below or above 5 kHz (Table 2). When comparing the percentages of calls with mean peak frequencies below 32 kHz among treatment groups, no significant difference was detected (*t*_33_ = −0.978, *p* = 0.335). Considering durations below 0.3 s, there was likewise no difference (*t*_33_ = 1.996, *p* = 0.054). The same was true for frequency modulations, since similar percentages of calls were emitted with modulations smaller than 5 kHz in both groups (*t*_33_ = 0.979, *p* = 0.335).

**TABLE 2:** Scatter plot distributions for Sal- and Halo-treated rats.

Sal *N* = 15/24			Mean peak frequency		
			=32 kHz	>32 kHz	Total calls
	Total numbers (percentages) means ± SEM		1,490 (93.4%) 72.77 ± 8.78	106 (6.6%) 27.23 ± 8.78	1,596 (100%)
Duration	<0.3 s	1,044 (65.4%) 59.39 ± 5.91	960 (60.2%)	84 (5.3%)	
	>0.3 s	552 (34.6%) 40.61 ± 5.91	530 (33.2%)	22 (1.4%)	
Modulation	<5 kHz	1,147 (71.9%) 60.27 ± 6.73	1,098 (68.8%)	49 (3.1%)	
	>5 kHz	449 (28.1%) 39.73 ± 6.73	392 (24.6%)	57 (3.6%)	

**Halo** ***N* = 20/24**			**Mean peak frequency**		
			**=32 kHz**	**> 32 kHz**	**Total calls**

			2,021 (97.9%) 83.44 ± 6.78	43 (2.1%) 16.56 ± 6.78	2,064 (100%)
Duration	<0.3 s	919 (44.5%) 40.48 ± 6.89	893 (43.3%)	26 (1.3%)	
	>0.3 s	1,145 (55.5%) 59.52 ± 6.89	1,128 (54.7%)	17 (0.8%)	
Modulation	<5 kHz	1,633 (79.1%) 50.76 ± 6.73	1,624 (78.7%)	9 (0.4%)	
	>5 kHz	431 (20.9%) 49.24 ± 6.73	397 (19.2%)	34 (1.6%)	

*Mean peak frequencies < or > 32 kHz, Durations = or > 0.3 s, frequency modulations = or > 5 kHz.*

**FIGURE 13:**
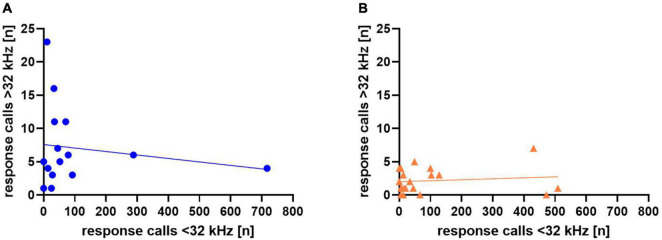
Correlation between calls <32 kHz and >32 kHz for Sal- **(A)** and Halo-treated **(B)** rats. Each data point represents response calls below and above 32 kHz of one animal.

In addition, we again asked whether response calls below or above 32 kHz were related in individual animals (Figure 13), but found no significant correlations in Sal- (*r* = −0.161, *p* = 0.566) or Halo-treated rats (*r* = 0.123, *p* = 0.606).

#### Relationships Between Response Calls and Playback-Induced Approach

To see whether social approach was associated with the emission of response calls, these two parameters were again correlated. The results were the same in both treatment conditions. In Sal-treated rats, there were no significant correlations, neither between the time spent in the proximal arms close to the active speaker nor between the entries into those with the amount of response calls (Sal time: *r* = −0.0195, *p* = 0.487; Sal entries: *r* = 0.059, *p* = 0.783). In Halo-treated animals, likewise no significant correlations between proximal time or entries and number of emitted calls were detected (Halo time: *r* = 0.143, *p* = 0.547; Halo entries: *r* = −0.112, *p* = 0.602). Moreover, locomotor activity during 50-kHz USV playback in comparison to the distance traveled before playback was not correlated with the total numbers of response calls, irrespective of treatment condition (Sal: *r* = −0.101, *p* = 0.639; Halo: *r* = −0.113, *p* = 0.598). In addition, locomotor activity during the first 15 min on the platform was not correlated with the number of response calls (Sal: *r* = −0.224, *p* = 0.421; Halo: *r* = 0.238, *p* = 0.312).

## Discussion

In this study, we characterized response calls emitted by rats exposed to playback of appetitive 50-kHz USV, previously shown to function as social contact calls (Wöhr, 2018). The phenomenon that rats respond to playback of species-specific 50-kHz calls by emitting response calls has been repeatedly reported before, but has not been described in detail yet (Wöhr and Schwarting, 2007, 2009; Willadsen et al., 2014; Willuhn et al., 2014; Engelhardt et al., 2017, 2018; Berg et al., 2018, 2021; Kisko et al., 2020; Olszyński et al., 2020, 2021; for an overview see Supplementary Table 1). First, we described the emission of response calls in reaction toward 50-kHz USV playback in WI rats. Secondly, we compared these to SD rats. Thirdly, we analyzed the effect of blocking DA receptors on response calls using Halo, as compared to vehicle-injected WI subjects.

Through these means, we could demonstrate that most rats emitted response calls. Importantly, the emission of response calls was clearly linked to 50-kHz USV playback. In fact, response calls were seen specifically in response to 50-kHz USV but not in response to time- and amplitude-matched noise, replicating previous results (Willadsen et al., 2014; Willuhn et al., 2014; Engelhardt et al., 2017, 2018; Berg et al., 2018, 2021; Kisko et al., 2020; Olszyński et al., 2020, 2021). When exposed to 50-kHz USV, receiver rats often started emitting response calls within the first minute of playback and emission rates were typically peaking after around 2–3 min, often outlasting playback for up to 5 min. This certainly supports naming these calls “response calls.”

Most response calls were characterized by peak frequencies below 32 kHz, the threshold typically applied to differentiate between 22- and 50-kHz USV (Brudzynski, 2001). Although peak frequencies were highly variable and ranged roughly between 20 and 80 kHz, the vast majority of response calls occurred in a frequency range of 20–32 kHz. Similarly, call durations were characterized by large variability, ranging from a few milliseconds to up to 1.5 s. Call durations of about 0.3 s occurred at a particularly high rate. Frequency modulations were typically below 5 kHz. When comparing these values to the parameters of typical 22- and 50-kHz USV, our values correspond more to 22-kHz USV; more precisely the short 22-kHz USV type since the durations were rarely longer than 0.3 s (Brudzynski, 2021).

The emission of response calls was seen in WI and SD rats, suggesting that this is a robust phenomenon not dependent on stocks. Specifically, we found that there were no substantial differences between WI and SD rats, concerning numbers of emitted calls, latencies to start calling, and call likelihood. In both stocks there was a large variability among response calls. However, their mean peak frequencies, call durations, and frequency modulations did not differ significantly between experimental conditions. SD rats only differed in one aspect by clearly showing calls around frequencies of 50 kHz, which was not that prominent in WI rats. This is somehow in line with other studies that also showed higher emission of 50-kHz USV and elevated rough-and-tumble play behavior in SD compared to WI rats (Manduca et al., 2014). Other studies, however, found that WI rats emitted more 50-kHz USV compared to SD rats (Schwarting, 2018a,b), indicating that WI rats are more prone to emit USV in general, which is also not represented by our data. If at all, on a descriptive level, WI rats emit slightly less response calls compared to SD rats. Regarding call parameters, previous studies showed marginal differences between stocks, i.e., shorter call durations in SD rats compared to WI rats (Schwarting, 2018b). On a descriptive level again, this aligns with our results, albeit this difference in call duration did not yield significance. Apart from stock differences, various other factors like breeding or experience have to be taken into account. Moreover, inter-individual differences should not be neglected, as our results also suggest (Schwarting, 2018a,b).

In our study, the pharmacological treatment with the D2 antagonist Halo did not affect call likelihood, call rates, latencies, temporal distribution, peak frequency, and frequency modulation. In Sal-treated WI rats, the majority of calls was again below 32 kHz, however, in Halo-treated rats this was even more prominent and Halo treatment also led to longer call durations. Previous studies showed that exposure to 50-kHz USV playback under the influence of systemically applied amphetamine, a catecholaminergic agonist, resulted in response calls with frequencies around 50 kHz at the expense of 22 kHz (Engelhardt et al., 2017). Specifically, calls of lower frequencies decreased drastically under the influence of amphetamine. In contrast, response calls in the 50 kHz range increased dose-dependently following amphetamine administration. This is in line with a large number of studies showing that the emission of 22- and 50-kHz USV are associated with the activation of distinct neurotransmitter systems (for review: Brudzynski, 2021). While 22-kHz USV are associated with the cholinergic system (Brudzynski, 2001; Kroes et al., 2007; Willadsen et al., 2018), the dopaminergic system plays an important role in the regulation of 50-kHz USV (Wöhr, 2021). For instance, electrolytic or 6-hydroxydopamine lesions of the ventral tegmental area reduce the emission of 50-kHz USV (Burgdorf et al., 2007). Conversely, emission of 50-kHz USV can be evoked by electrical stimulation of the ventral tegmental area or the nucleus accumbens (Burgdorf et al., 2000, 2007). Moreover, psychostimulants, most notably amphetamine, lead to a robust increase in 50-kHz USV emission (Rippberger et al., 2015). Additionally, playback of 50-kHz USV was shown to induce enhanced levels of activity in the nucleus accumbens (Sadananda et al., 2008), where it elicits a rapid phasic release of dopamine (Willuhn et al., 2014). Based on these findings, one could have assumed that the dopaminergic receptor blockade with Halo should decrease response call numbers, especially those above 32 kHz, which was apparently not the case. Possibly, these calls are not critically dependent on dopamine D2 receptor function, and might be dependent on endogenous opiates, as indicated by an earlier playback study with the opiate receptor antagonist naloxone (Wöhr and Schwarting, 2009).

Together, the present findings indicate that the emission of response calls is a robust phenomenon that is seen specifically in response to playback of 50-kHz USV independent of stock and despite blocking dopamine neurotransmission. These observations are in line with the idea that the emission of response calls reflects changes in affect that are caused by playback of 50-kHz USV. For example, one might expect the induction of a positive affective state in response to appetitive 50-kHz USV. On the other hand, it was suggested that response calls reflect frustration induced by the inability to reach the conspecific emitting 50-kHz USV. Alternatively, response calls might serve communicative functions as social contact calls or as appeasement signals. While the present findings do not allow drawing strong conclusions about causes and functional significance of response calls, they provide first insights into potential mechanisms underlying their emission.

In support of the idea that response calls might reflect an affective state we hypothesize that the rats are not solely in one affective state, but rather in an ambivalent state. There is convincing evidence in support of the notion that USV emission reflects prominent affective states (Brudzynski, 2021) and that different call types are associated with distinct states (Brudzynski, 2013b). Because USV below 32 kHz are typically believed to function as alarm or distress calls reflecting a negative affective state, this would suggest that playback of 50-kHz USV induced a negative state in the receiver rats. However, the strong level of social approach behavior and the emission of 50-kHz response calls, at least in SD rats, evoked by playback of 50-kHz USV speaks against the induction of a solely negative affective state through 50-kHz USV playback (Wöhr, 2018). Furthermore, the positive and negative emotional states in rats were proposed to be mutually exclusive and acting in an antagonistic manner (Brudzynski, 2021). It is possible, however, that the two states quickly alternate which leads to the hypothesis of an ambivalent state, with negative and positive phases present in an oscillating manner. This is also reminiscent of an approach/avoidance conflict, i.e., a situation characterized by choices leading to either reward or punishment (Aupperle et al., 2015). Interestingly, it was shown that rats emit 22-kHz as well as 50-kHz USV during neutral situations and not only aversive ones (Robakiewicz et al., 2019). The study by Robakiewicz et al. (2019) also showed that both call types and hence presumably both emotional states can be present during an emotional neutral task of performing nose pokes in order to change the light of the experimental apparatus. Both call types were also found in a cocaine self-administration task (Barker et al., 2010), where animals received either high or low doses of cocaine. Low dose rats predominantly emitted short 22-kHz calls and high dose rats emitted mostly 50-kHz calls. Nevertheless, both groups showed calls of both emotional states and this supports the hypothesis of the ambivalent state. In the present study, however, only SD rats emitted 50-kHz USV to a higher extent and all other experimental groups mainly emitted calls with frequencies below 32 kHz. Additionally, the emissions of response calls below and above 32 kHz were not correlated across individual rats, suggesting that there was no general tendency for emitting response calls in both frequency ranges, which speaks against the hypothesis of an ambivalent state.

With respect to the emission of 22-kHz calls, this phenomenon might be explained by the hypothesis of a frustrated state in the receiver rat, possibly induced by the violated expectation of another rat being present. Other studies suggested that short 22-kHz calls (<0.3 s) represent a dysphoric state or displeasure without any external threat (Simmons et al., 2018), which is in line with the mean peak frequencies, durations, and low frequency modulations of the response calls found in our study. This might also be an indication that calls with low frequencies in response toward 50-kHz USV playback are an expression of internal distress, i.e., frustration, as suggested before (Wöhr and Schwarting, 2009). Frustration is defined as a result of behavior after an expected but not received reward (Scull et al., 1970; Burokas et al., 2012). In our playback paradigm, the rat probably realized that there was no rat physically present for interaction after hearing the 50-kHz USV playback, and this could have led to a state of frustration in the approaching rat. This might also explain why the majority of response calls was emitted within 2 or 3 min after the onset of the 50-kHz USV playback. At first, the animals heard and recognized the stimulus, exhibited a strong social approach immediately afterward and as soon as the rats realized that there was no conspecific present, the emission of response calls increased as an expression of a frustrated state. In line with the frustrated state hypothesis is our finding that the first calls of most animals of data set 2 and 3 were of higher frequencies, i.e., around 50 kHz and quickly changed to calls with frequencies in the 22-kHz USV range (Supplementary Figure 1).

On the other hand, the positive correlation of response calls and approach behavior might serve the hypothesis that the response calls could also be characterized as social contact calls. 50-kHz USV have been postulated to fulfill an affiliative communication function to, for example, maintain a playful state during rough-and-tumble play or as social contact calls to reestablish social proximity after separation of conspecifics (Wöhr et al., 2016). An indication that the response calls in our study serve as social contact calls is that they are emitted during social approach behavior. Further, such calls are emitted frequently during the approach behavior like 50-kHz USV during rough-and-tumble play (Knutson et al., 1998). In our study we found a moderate positive correlation between response calls and approach behavior, i.e., the time spent close to the active speaker, in SD and, at least to some extent, in WI rats. Apparently, the more the animals tried to reach a possible conspecific signaled by the 50-kHz USV playback, the more calls they emitted, supporting the hypothesis of response calls being contact calls. For Sal- and Halo-treated WI rats, however, this was not the case. In Halo-treated rats, the absence of a positive correlation between approach behavior and response call emission was probably due to the drug-induced immobility (Berz et al., 2021). Since Sal-treated rats also received an i.p. injection 60 min prior to testing, this might have influenced their approach response, as well as their calling behavior; even though Sal-treated rats significantly approached the sound source (Berz et al., 2021) and emitted similar numbers of response calls as WI rats. No correlation was found, however, between overall activity and call numbers in any group. Also or alternatively, the positive correlation between approach behavior and response calls especially observed in SD rats might not be in order to establish contact, but rather due to hypervigilance. Olszyński et al. (2021) showed that in response to 50-kHz USV playback, heart rate and locomotor activity increased as well as the emission of USV. The USV in response to 50-kHz USV playback in that study were mainly 50-kHz calls, possibly representing contact calls, in contrast to our study here, where the animals mostly emitted calls of lower frequencies. Also, the peak of call emission occurred shortly after the recipient of the playback was in proximity to the sound source and ceased after playback has stopped, which suggests that these calls could function to establish social contact or in search of it. However, the response calls linked to the 50-kHz USV playback do not classify as 50-kHz calls because their mean peak frequencies are much lower, the duration is longer, and there is hardly any frequency modulation compared to 50-kHz calls.

Alternatively, response calls could serve appeasing purposes. The age difference between the rat of the recorded playback and the test subject might be of interest, because in our study, a juvenile rat heard 50-kHz USV playback recorded from an adult rat and accordingly, it seems plausible for the subject rat to cautiously approach the potential conspecific. Supporting this hypothesis, is the fact that in adult male rats, USV calls of lower frequencies were found during play fighting (Burke et al., 2017, 2020). In social situations that were at risk to escalate into aggression, the play partners lowered their calls gradually from 50 kHz to around 30 kHz with increasing durations (Burke et al., 2017). The authors hypothesized that this group of calls might be a transition from 50-kHz flats to 22-kHz flats or a unique new type of calls. The function of these calls is probably the induction of appeasement, i.e., to de-escalate a situation at risk to turn into aggression (see also Sales, 1972; Lore et al., 1976). Our results seem to support this hypothesis since we tested juvenile rats subjected to calls from an older adult rat and the response calls were in similar frequencies. Moreover, the response calls had also similar frequency modulations, like the calls in the study by Burke et al. (2017) and were not exclusively flat as the common 22-kHz USV. So far, however, it is not known whether receiver rats can gain information about the age of the sender based on their USV.

Importantly, the response call phenomenon studied here in detail appears sufficiently robust to be used as a measure for the reciprocal nature of acoustic communication and can easily be applied in rat model systems for neuropsychiatric disorders, where acoustic communication is impaired, such as autism spectrum disorder (Lai and Baron-Cohen, 2015). In preclinical studies examining USV with the aim to reveal communication deficits in rodent model systems, most laboratories have focused exclusively on the sender. Although there is now an increasing number of preclinical studies including playback paradigms to learn about the responses evoked in the receiver as well (Berg et al., 2018, 2020a,b; Kisko et al., 2018, 2020; Wöhr et al., 2020), an important aspect of acoustic communication that is often still neglected is its reciprocal nature and the fact that a signal emitted by the sender frequently evokes the emission of a response signal in the receiver (Seyfarth and Cheney, 2003). Measuring response calls offers a unique opportunity to overcome this limitation. It offers a new approach to studying the reciprocal nature of communication in rodent models for neuropsychiatric disorders.

## Data Availability Statement

The raw data supporting the conclusions of this article will be made available by the authors, without undue reservation.

## Ethics Statement

The animal study was reviewed and approved by Tierschutzbehoerde, Regierungspraesidium Giessen, Germany, TVA No. 35-2018.

## Author Contributions

RS and MW designed the study, acquired resources and funding, and oversaw the project. AB performed the experiments. AB with substantial help from MW analyzed the data. AB, RS, and MW wrote the manuscript. All authors contributed to the article and approved the submitted version.

## Conflict of Interest

The authors declare that the research was conducted in the absence of any commercial or financial relationships that could be construed as a potential conflict of interest. The handling editor declared a past co-authorship with one of the authors MW.

## Publisher’s Note

All claims expressed in this article are solely those of the authors and do not necessarily represent those of their affiliated organizations, or those of the publisher, the editors and the reviewers. Any product that may be evaluated in this article, or claim that may be made by its manufacturer, is not guaranteed or endorsed by the publisher.

## Footnotes

1
https://github.com/UEFepilepsyAIVI/DeepSqueak.git
