# Supplementary figures and images for: Response Calls Evoked by Playback of Natural 50-kHz Ultrasonic Vocalizations in Rats
Source: Front Behav Neurosci. 2022 Jan 14;15:812142. doi: 10.3389/fnbeh.2021.812142 (PMC8797927; doi:10.3389/fnbeh.2021.812142)

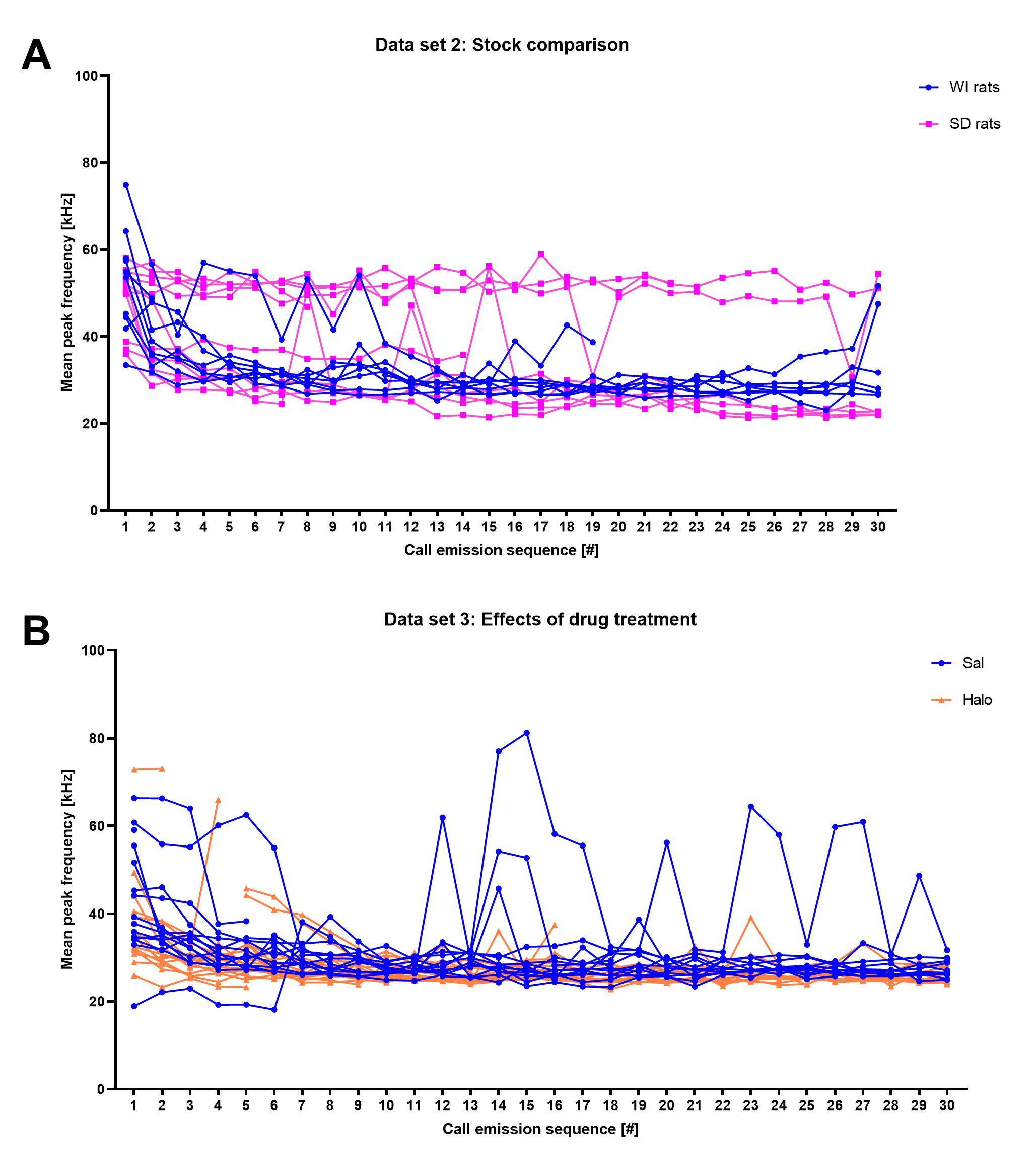

Supplement: Supplementary file 1 [file Image_1.JPEG]
